# Extracellular vesicles in phytopathogenic fungi

**DOI:** 10.20517/evcna.2023.04

**Published:** 2023-03-30

**Authors:** Brian D. Rutter, Roger W. Innes

**Affiliations:** Department of Biology, Indiana University, Bloomington, Indiana, IN 47405, USA.

**Keywords:** Fungal phytopathogens, extracellular vesicles, EVs

## Abstract

Extracellular vesicles (EVs) are nano-sized lipid compartments that mediate the intercellular transport of lipids, proteins, nucleic acids and metabolites. During infectious diseases, EVs released by host cells promote immune responses, while those released by pathogens attempt to subvert host immunity. There is a growing body of research investigating the role of fungal EVs in plant pathosystems. It is becoming clear that EVs released by fungal phytopathogens play a role during infection through the transport of protein effectors, toxic metabolites and RNA. Here, we discuss recent findings on EVs in fungal phytopathogens, including the methods employed in their isolation, their characterization, contents and functionality, as well as the key questions remaining to be addressed.

## INTRODUCTION

Cells in all domains of life release nano-sized, membrane-bound compartments called extracellular vesicles (EVs). Though initially considered little more than cellular waste^[1]^, EVs are now appreciated as potent mediators of intercellular communication^[2]^.

There are generally three subclasses of EVs, grouped according to their form of biogenesis. Apoptotic bodies (ABs), which are usually the largest (≥ 1,000 nm in diameter) and most heterogeneous, are the product of membrane blebbing during programmed cell death. Microvesicles (MVs, also known as ectosomes or microparticles; ~100-1,000 nm in diameter) bud directly from the plasma membrane of living cells. Lastly, exosomes (~30-150 nm in diameter) form inside living cells as a result of membrane invaginations into the lumen of a late endosome; they are released after the fusion of a late endosome with the plasma membrane^[3]^. Overlapping size ranges and protein markers among these classes of vesicles make it extremely difficult to classify isolated EVs as exosomes, microvesicles or ABs. In the absence of direct observations of biogenesis or highly specific markers, EVs are more often categorized according to physical characteristics, such as size, buoyant density or biochemical composition^[4]^.

Once released from a cell, these extracellular organelles provide a protective environment for the long-distance delivery of proteins, lipids, nucleic acids and other bioactive compounds. EVs exert their effects on recipient cells through interactions with surface receptors or the transfer of EV cargo through membrane fusion or endocytosis^[2]^. This form of long-distance communication is not limited to cells within an organism or of the same species but can occur across species and even kingdoms^[5]^.

The ability of EVs to transfer molecules across kingdoms is particularly important during infectious diseases, where both the host and pathogen secrete vesicles in an effort to promote or subvert immunity, respectively^[6]^. In mammals, various cell types release EVs in response to stress and disease. Vesicles released under these conditions are capable of mediating coagulation, promoting or suppressing inflammation and modulating adaptive immune responses through the transfer of antigens^[6-8]^. In some cases, they may even directly inhibit the growth and spread of invading microbes^[9]^.

Pathogens, such as viruses, bacteria, fungi and protozoan parasites, also utilize EVs during infections, either releasing their own vesicles or inducing host cells to secrete EVs containing pathogen-derived molecules^[10,11]^. These vesicles can be a two-edged sword. On the one hand, components of pathogen-derived EVs are often recognized by the host as pathogen-associated molecular patterns and inadvertently trigger immune responses^[10,11]^. On the other hand, pathogen-derived EVs can contain toxins and molecules with immunity-modulating properties that enhance pathogen survival, infectivity and spread^[10,11]^.

EVs can also benefit pathogens in ways that do not directly interfere with the host immune system. For example, both bacteria and single-celled fungi produce vesicles that enhance the formation of biofilms and increase antibiotic resistance^[12-14]^. Moreover, highly virulent strains of bacteria can stimulate less virulent strains to heightened states of infectivity through an EV-mediated exchange of virulence factors, including proteins and genetic material^[15-17]^.

While the overwhelming majority of research into host- and pathogen-derived EVs concerns human diseases, the tit-for-tat exchange of vesicles during infections is by no means limited to mammalian systems of pathology. EVs also play a prominent role in plant pathosystems. Electron microscopy studies have revealed the presence of exosome-like vesicles secreted underneath sites of pathogen attack and around invading fungal tissues^[18-21]^. Many of these vesicles are clearly generated by the plant, but other EVs may be pathogen-derived^[21]^. Isolated plant EVs are enriched for proteins involved in stress and defense responses and secreted in greater abundance in response to stress hormones and bacterial infections^[22]^. Plant EVs have also been shown to have direct, cytotoxic effects on fungal spores^[23,24]^.

In turn, plant-pathogenic bacteria and fungi produce their own EVs loaded with cytotoxic compounds and immunity-modulating virulence factors^[25]^. While research into these pathogen-derived EVs has been limited, there is a growing recognition of their importance for understanding and better managing plant diseases. This is particularly true of EVs produced by phytopathogenic fungi, owing to the massive economic and environmental impact of these pathogens and the increased threat of their spread due to elevated global temperatures^[26]^. The purpose of this review is to present the current state of knowledge on phytopathogenic fungal EVs, how they are isolated, which molecules are associated with them and what role they play during plant infections.

## A BRIEF HISTORY OF FUNGAL EVs

While fungal EV research may seem relatively new, evidence for EVs in fungi has existed for decades. In fact, the first published images of EV secretion came from a study of the wood-decaying fungus *Polystictus versicolor*^[27]^. Early on, vesicle-like structures between the plasma membrane and cell wall of fungal hyphae were referred to as “lomasomes” or “border bodies”. They were thought to be unique to fungi and contribute to the formation of the cell wall^[28]^.

Over the years, multiple studies documented the presence of EVs in fungi using electron microscopy. Microvesicle secretion was observed in protoplasts of *Aspergillus nidulans*^[29]^, while evidence for exosome release was observed in freeze-fractured cells of *Cryptococcus neoformans*^[30]^. Moreover, in *Candida tropicalis*, EVs were observed traversing the cell wall^[31]^, accumulating in the liquid growth medium in the presence of n-alkanes^[32]^ and adhering to the surface of fungal protoplasts undergoing cell wall regeneration^[33]^.

Despite these many observations, fungal EVs were not officially isolated until 2007, when Rodrigues *et al*. pelleted EVs from liquid cultures of *Cryptococcus neoformans*, a common environmental yeast and opportunistic human pathogen^[36]^. These vesicles were associated with several virulence-related molecules, including a major capsular polysaccharide, pigment and proteins^[34-36]^.

Virulence-related molecules appear to be a common feature of fungal EVs, as subsequent studies examining different species have shown^[37-40]^. The presence of such molecules suggests that fungal EVs play a role in pathogenesis, and a number of findings support this hypothesis. For example, EVs from *Cryptococcus neoformans* and *Sporothrix brasiliensis* enhance the spread of each respective fungus throughout its host^[41,42]^, while EVs from *Candida albicans* can promote the formation of antibiotic-resistant biofilms^[12]^. Through cell-to-cell communication, EVs from virulent strains of *Cryptococcus neoformans* and *Cryptococcus gattii* can also enhance the proliferation and survival of less virulent strains^[15,43]^.

While fungal EVs can benefit pathogens, they can just as easily work to their detriment. Such vesicles are often immunogenic, able to induce inflammation and stimulate the antimicrobial activities of host macrophages^[44-46]^.

To date, EVs have been isolated from over twenty species of fungi, including both yeasts^[37,39-41,43,46-50]^ and filamentous fungi^[24,51-62]^. The majority of these fungi cause disease in humans, a few are valued for their applications in industry, and a small but growing list are phytopathogens. The latter list of fungi includes: *Alternaria infectoria*, the causative agent of wheat black point^[60]^, *Fusarium oxysporum* f. sp. *vasinfectum *(*Fov*), which causes vascular wilt disease in cotton^[52,62]^, *Fusarium graminearum*, which causes Fusarium stalk rot in cereal crops^[24,55]^, *Zymoseptoria tritici*, which causes Septoria Tritici Blotch in wheat^[56]^, *Ustilago maydis*, the causative agent of maize smut^[57]^, and *Colletotrichum higginsianum*, which causes anthracnose disease in cruciferous plants^[59] ^[Table 1].

**Table 1:** Characteristics of fungal phytopathogen EVs

**Species [Reference]**	**Transmission electron microscopy/** **Scanning electron microscopy**	**Dynamic light scattering/** **Nano-particle tracking**
*Alternaria infectoria* ^[60]^	Appearance = cup-shaped particles Range = 20-40 nm Mean diameter = 28.36 nm	Mean diameters = 50 nm and 100 nm
*Fusarium oxysporum* f. sp. *vasinfectum*^[52]^	Appearance = cup-shaped and multi-lobed rosette-shaped particles	Mean diameter = 155.1 nm Mode diameter = 150.0 nm
*Fusarium oxysporum* f. sp. *vasinfectum*^[62]^	Appearance = spherical and cup-shaped particles	Range = 100-300 nm Mean diameter = 120.0 nm
*Fusarium graminearum* ^[55]^	Appearance = cup-shaped particles	Mean diameter = 120 nm
*Fusarium graminearum* ^[24]^	Appearance = cup-shaped particles	Mean diameters = 200 nm, 123.8 nm and 232.9 nm Mode diameter = 93.6 nm, 94.2 nm and 115.2 nm
*Zymoseptoria tritici* ^[56]^	Appearance = cup-shaped particles Range = 50-300 nm Mean diameter = 91.8 nm Median diameter = 84.0 nm	Range = 100-250 nm
*Ustilago maydis* ^[57]^	Appearance = cup-shaped particles	N/A
*Colletotrichum higginsianum* ^[59]^	Appearance = cup-shaped particles	Mean diameters = 106 nm and 100-102 nm Median diameters = 104 nm and 100-102 nm

## METHODS OF ISOLATION AND PURIFICATION

Vesicle isolation is the first and most serious challenge in any EV study. Choices made early in the methodology can have significant impacts on a study’s findings, and variability among laboratories can lead to conflicting data. International consortiums of EV researchers have made great efforts to standardize the implementation and reporting of methods^[4,63]^. Still, when dealing with multiple species, as is the case with fungal EV research, variability is inescapable.

**Figure 1:**
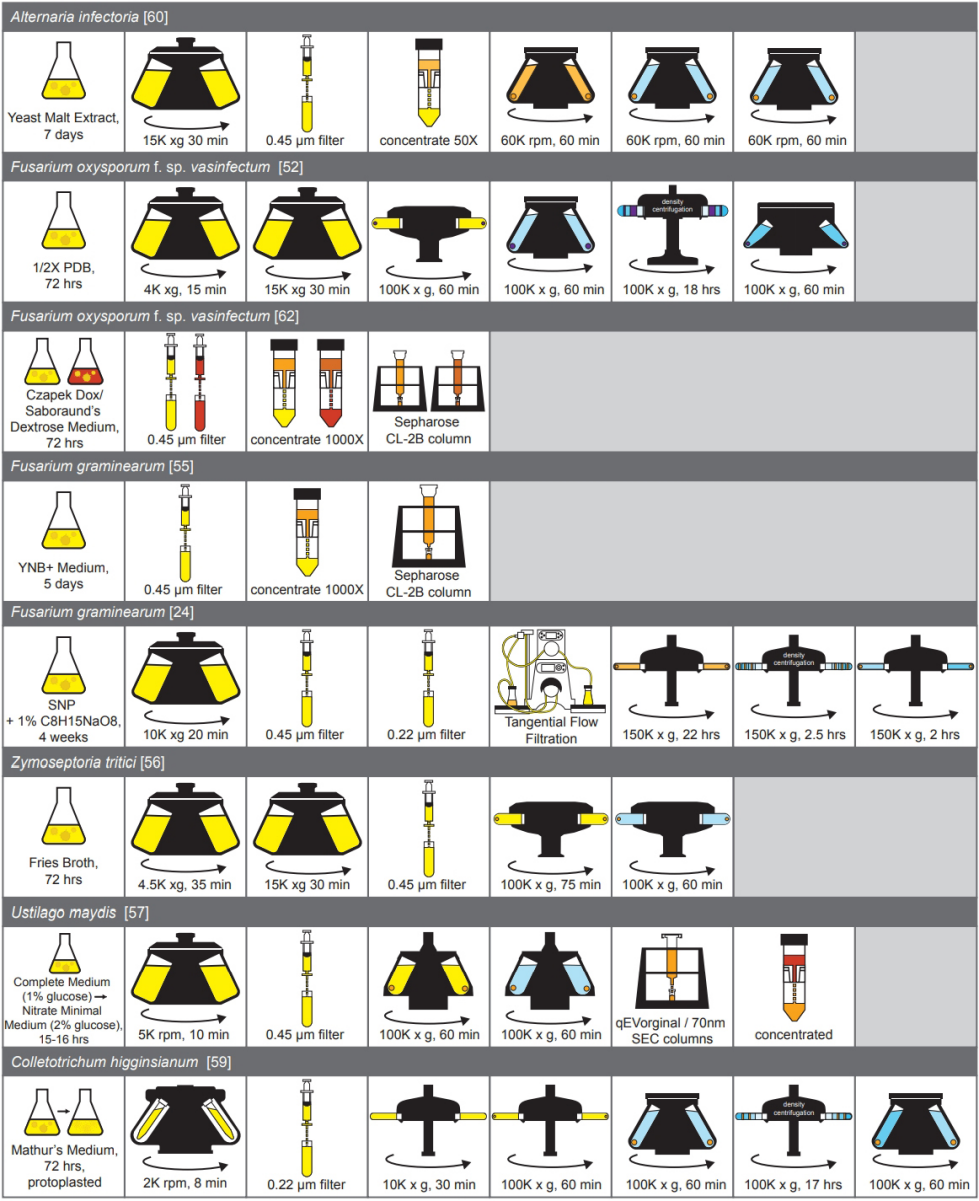
A Comparison of EV Isolation Protocols for Fungal Phytopathogens. To isolate fungal phytopathogen EVs, fungi are first grown in liquid cultures. The mycelial tissues are pelleted or strained out and the supernatant is used to isolate EVs. In the case of *C. higginsianum*, mycelia are first incubated with hydrolyzing enzymes to remove cell walls. Differential ultracentrifugation is the most common technique for isolating EVs. The supernatant is first centrifuged at low-speed (10-15K x *g* for 30 min) to remove large particles and potential debris. Before or after low-speed centrifugation, it is common to pass the supernatant through a 0.45 or 0.22 µm filter. The supernatant is then subjected to high-speed centrifugation (100K x *g* for 60 min) to pellet EVs. Density-gradient ultracentrifugation (100-150K x *g* for 17-22 hrs) can be used to purify EVs away from contaminating particles and separate EVs into different populations. All centrifugation steps are performed at 4 ℃. Other studies use size exclusion chromatography to isolate fungal EVs from the culture supernatant. This technique can be used alone or in combination with differential ultracentrifugation.

The majority of fungal EV researchers have adapted their methods from Rodrigues *et al*., which used differential ultracentrifugation (DUC) to isolate EVs from the supernatants of liquid cultures^[36] ^[Figure 1]. These methods remain the gold-standard approaches for fungal EV isolation despite their limitations. DUC, by its nature, is time-consuming, can require large volumes (hundreds of milliliters to liters) of culture and often leads to significant losses in the yield of EVs.

At the very beginning, fungi are grown in a liquid culture for several days. There is no standard medium when growing phytopathogens for EV isolation. Studies have used a range of both natural and synthetic broths that can be either complete or minimal in composition [Figure 1]. Because the EVs reflect their cell of origin, differences in the nutritional content of growth media can lead to significant alterations in EV composition across studies. For example, when grown in two different kinds of media, a defined medium and a nutrient-rich tissue lysate, *Fov* produced similar numbers of EVs but with different protein cargos^[62]^. A similar observation was made for the yeast and human pathogen *Histoplasma capsulatum*, the EVs of which were altered in both protein and lipid compositions in response to nutrition^[64]^. It is unknown whether these changes merely reflect an altering of EV composition or a shift in the release of different EV populations.

Another complexity of growing filamentous plant pathogens is the presence of macro-morphologies. Yeast grows into uniformly dispersed cultures, but filamentous fungi develop large aggregates known as mycelial clumps and pellets (not to be confused with the kind of pellet produced after centrifugation)^[65]^. Currently, it is unknown whether these dense and sometimes irregular structures affect the diffusion of EVs into the medium. In our own experience, breaking down these structures using cell wall-degrading enzymes drastically improves EV isolation; however, this may also signify that the cell wall is itself a significant barrier to fungal EV isolation^[59]^. If grown for too long, mycelial pellets may contribute to cellular contamination in EV preparations. The inner core of these pellets is dense and allows for poor oxygen diffusion. Eventually, cells in the core undergo apoptosis, which could taint EV preparations with cytoplasmic contamination^[65]^.

Depending on the organism, fungal phytopathogens have been grown from 16 h to 4 weeks, although 72 h is the most common period [Figure 1]. Fungal tissues in the culture are pelleted at 4,000 to 8,000 rpm and the supernatant is processed for EVs. One low-speed spin (~10,000 to 15,000 x g lasting 30 minutes) is used to pellet any large particles or potential cellular debris [Figure 1]. The supernatant can then be concentrated using an ultrafiltration system and the EVs pelleted at 100,000 x g for one hour [Figure 1]. Some studies also include filtration through a 0.22-0.45 µm filter before or after the low-speed centrifugation step, as well as multiple washes of the EV pellet [Figure 1].

Yields for DUC are typically low, which requires some laboratories to pool multiple samples to obtain enough material for analyses^[52]^. Using size exclusion chromatography (SEC) instead of DUC, Garcia-Ceron *et al*. was able to double or even triple the amount of EVs isolated from *Fov* cultures compared to experiments using DUC^[62]^. SEC allows large volumes of supernatant to be processed for EVs while better preserving their integrity by avoiding centrifugal forces that could damage vesicles or cause aggregates. Another method for improving yield is to generate protoplasts. For example, little to no EVs were found in the culture supernatant of *C. higginsianum*. Only when the cell walls were digested could EVs be isolated^[59]^. This technique has also been used to effectively isolate EVs from the filamentous, opportunistic human pathogen *Aspergillus fumigatus*^[66]^. Although it improves yield, the more fragile nature of protoplasts means a higher risk of cytoplasmic contamination. Removing the cell wall could also influence the protein cargo of EVs as the fungus responds to stress and attempts to generate new cell walls^[66]^.

EV pellets are heterogeneous in nature, containing both different populations of EVs and non-vesicular components. Purification is encouraged when attempting to define a biomarker for EVs or determine functionality^[4]^. SEC can be used in tandem with DUC as a way to purify EV pellets away from non-vesicular components^[57]^. This method is fairly successful at removing non-vesicular components but cannot completely eliminate some soluble proteins and lipoproteins^[67,68]^. Density gradient ultracentrifugation using sucrose or iodixanol, an iso-osmotic compound with low toxicity, is another common technique for purifying EV samples^[24,52,59]^. This method is more successful at isolating EVs away from non-vesicle components but can reduce yield^[68]^. Both SEC and density gradient ultracentrifugation have the added benefit of being able to separate different populations of vesicles^[24,59,67]^.

## EV CHARACTERISTICS

After isolation, EV pellets are characterized to confirm the presence of membrane vesicles. Common analyses include negative staining transmission electron microscopy (TEM), dynamic light scattering (DLS) or nano-particle tracking (NTA) and, when possible, immunoblots for protein EV markers.

Fungal EVs can range from 20 to 1,000 nm in diameter^[69]^. When negatively stained for TEM, EVs from fungal phytopathogens appear as cup-shaped objects ranging from 50 to 500 nm in diameter^[55,56] ^[Table 1]. NTA of EVs from fungal phytopathogens has recorded diameters ranging from ~90 to 300 nm^[24,52,55,56,59,62]^, while DLS of EVs from *Alternaria infectoria* recorded two populations of EVs, one with a diameter of 50 nm and the other with a diameter of 100 nm^[60] ^[Table 1]. These slightly different measurements no doubt depend upon the species of fungi and the growth conditions, but they also have much to do with the method of measurement. TEM allows researchers to observe a wide range of particles, but measurements can be inaccurate owing to human error and shrinkage of particles that occurs while preparing samples. NTA struggles to measure particles smaller than 100 nm, and while DLS can detect particles over a much wider range (10 nm to 1 µm), it has less resolution than NTA.

Protein biomarkers are a common tool used to verify the presence of EVs in a pellet. For example, small (< 200 nm in diameter) mammalian EVs are enriched for specific proteins, including tetraspanins such as CD9 and CD81, which play a role in membrane organization^[70]^, and members of the endosomal sorting complex required for transport (ESCRT), such as Tumor Susceptibility Gene 101 (TSG101) and Alix, which are required for the budding of intralumenal vesicles into the interior of multivesicular bodies^[71,72]^. Unfortunately, there are currently no standard biomarkers for fungal EVs. While fungi encode proteins with a very similar structure to mammalian tetraspanins (i.e., with four transmembrane domains and a pattern of conserved cysteines in one of two extracellular loops), they do not possess true orthologs, and fungal “tetraspanins” have not yet been detected in any available fungal EV proteome^[73]^. Fungi do possess orthologs to ESCRT proteins, but these proteins are either not detected or present at very low levels in fungal EV proteomes^[52]^.

Work on *Candida albicans* identified 22 potential protein markers enriched in EVs compared to the whole cell lysate. The list includes three members of the Sur7 family^[74]^. Members of the Sur7 family possess four transmembrane regions, similar to tetraspanins, and, in *S. cerevisae*, function as members of a large complex associated with endocytosis^[75]^. Sur7 family proteins have since been detected in several fungal EV proteomes, including those of *Cryptococcus, Z. tritici* and *C. higginsianum*^[56,59]^.

Our research found that Sur7 family proteins were not abundantly present in *C. higginsianum* EVs and instead chose to focus on the v-SNARE *Ch*Snc1, the t-SNARE *Ch*Sso2 and the 14-3-3 protein *Ch*Bmh1^[59]^. Members of the Snc1, Sso1/2, and Sec9 SNARE complex function in vesicle docking and fusion at the plasma membrane^[76]^. Orthologous proteins have been linked to the unconventional secretion of effectors in the phytopathogens *Magnoportha oryzae* and *V. dahliae*^[77,78]^, as well as phytotoxin secretion in *F. graminearum*^[79]^. As a 14-3-3 protein, *Ch*Bmh1 facilitates protein-protein interactions. Orthologs of this protein have been detected in EVs from other phytopathogens, including *Fov* and *Z. tritici*^[52,56]^. 14-3-3 proteins and SNARES are also common components of plant EV proteomes^[22]^.

We could generate transgenic strains of fungi expressing fluorescently labeled versions of each protein. We further demonstrated that the fusion proteins were protected from protease degradation inside membrane vesicles^[59]^. *Ch*Snc1 and *Ch*Sso2 were also enriched in EVs compared to cell lysate, suggesting they may make excellent EV markers^[59]^.

Whether a universal marker for fungal EVs can be developed remains to be seen. Variability in EV protein cargo undoubtedly arises with different species of fungi, growth conditions and methods of isolation. More standardization of methods may be required before a universal fungal EV marker can be established.

## PROTEIN CONTENT

Fungal EVs are associated with a diverse range of proteins, including enzymes involved in metabolism, oxidation/reduction, cell wall synthesis, signaling, translation and transport^[80,81]^. Many of these proteins are sequestered from the cytoplasm, but a high proportion are associated with the plasma-membrane^[81]^. Additionally, because EV secretion represents a form of unconventional secretion, a lower percentage of EV proteins possess signal peptides.

Historically, identifying fungal EV proteins has been a struggle, with most published proteomes containing fewer than 100 proteins^[81]^. This is likely due to the challenge of isolating sufficiently high numbers of fungal EVs, as well as differences in methodology used in isolation and proteomic analysis. Despite these challenges, research into fungal phytopathogen EVs has produced EV proteomes with larger numbers of proteins, ranging from 420 to 710 proteins; the one exception being the EV proteome of *A. infectoria*, which contains only twenty proteins [Table 1]. To date, there are published proteomes for *A. infectoria*, *Fov*, *F. graminearum, Z. tritici *and *C. higginsianum*^[52,55,56,59,60,62]^.

**Figure 2:**
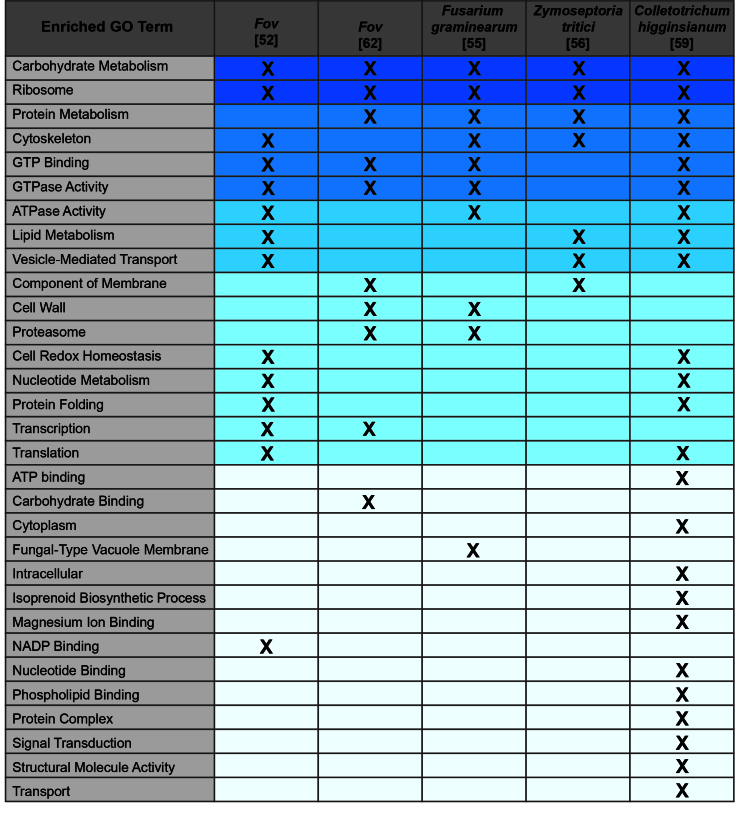
Common Enriched GO Terms Among Fungal Phytopathogen EV Proteomes.Enriched GO terms reported for fungal phytopathogens were compiled into a concise list.The presence of a GO term in the EV proteome of a fungus is indicated with an"X."Shading highlights the frequency of appearance across the five datasets;darker shades indicate GO terms that are present in more datasets.

When analyzed for gene ontology (GO) terms, fungal phytopathogen EV proteomes are enriched for proteins involved in general metabolism, translation, the cytoskeleton, GTPase/ATPase binding/activity, vesicle-mediated transport, cell redox homeostasis, protein folding and regulation of transcription, as well as proteins associated with the membrane and cell wall [Figure 2]. The percentage of proteins with predicted transmembrane regions varies with the species and the growth conditions and ranges from ~6%-35%^[52,55,56,59,62]^. The same is true for proteins with signal peptides, ranging from ~6%-30%^[52,55,56,59,62]^.

A common feature of EVs from fungal phytopathogens is the presence of virulence-related proteins, especially those involved in the production of secondary metabolites (SMs). Proteins belonging to SM-producing biosynthetic gene clusters have been identified in EVs isolated from every phytopathogen tested so far, with the exception of *Z. tritici*. Such proteins are often enriched in vesicles compared to the whole cell lysate and include polyketide synthases for the production of melanin in *A. infectoria*, fusarubin in *Fov* and higginsianins in *C. higginsianum*^[52,59,60,62]^. Enzymes for the production of mycotoxins are also present in *Fov* EVs^[52,62]^, while EVs from *F. graminearum* are associated with several SM-related proteins, including those involved in the synthesis of aflatoxins^[55]^. These metabolites not only contribute to the virulence of the fungus on its host plant, but their presence in food can have damaging effects on livestock and human health^[82,83]^.

It has been suggested that SM-related proteins may allow fungal EVs to function as “mini-factories” for the production and delivery of toxins^[55]^. Indeed, SMs have been identified in association with fungal phytopathogen EVs. A purple pigment co-fractionated with *Fov* EVs presents an iodixanol density gradient. This pigment possibly stimulates cell death in cotton leaves and, due to the presence of a fusarubin cluster-esterase in *Fov* EVs, it was initially thought to be purpurfusarin^[84]^ or 8-O-methyl anhydrofusarubin^[82]^. However, the pigment’s UV-vis absorbance spectra better match a naphthoquinone ^[52]^. The same study also detected a polyketide bikaverin, which has known antibiotic properties and may indicate that fungal EVs play a role in microbial competition^[52,85]^. Still, it is unknown whether these metabolites are merely associated with fungal EVs, packaged inside of them before their release or synthesized in vesicles after their release.

Proteases and cell wall modifying proteins are also present in fungal phytopathogen EVs and may contribute to virulence. Proteases are some of the most abundant proteins in *Fov* EVs and have also been detected in EVs from *F. graminearum* and *C. higginsianum*^[52,55,59,62]^. Proteins involved in cell wall maintenance and modifications, such as chitinases, glucanases and a variety of synthases, are equally abundant^[52,55,59,60,62]^. Together, these enzymes may promote penetration into host tissues or facilitate the release of EVs past the fungal cell wall^[62]^. The presence of cell wall-associated enzymes may also indicate that fungal EVs play a role in the maintenance of the cell wall, as has been shown for *Aspergillus fumigatus* and *S. cerevisiae*^[66,86]^.

Other virulence-related proteins include oxidoreductases, which could protect fungi from reactive oxygen species produced early on during the plant immune response. Putative effectors have also been detected in EV samples. Some of these effectors are small cysteine-rich proteins, a common source of proteinaceous effectors in phytopathogenic fungi^[87]^. Other proteins are homologous to known effectors in other species of phytopathogens or possess domains commonly found in known effectors, such as LysM, NPP1 or AltA-1^[55,62,88,89,90]^. Although many of these effectors have yet to be verified, some putative EV-associated effectors in *F. graminearum* were shown to have high expression levels during infection on maize, suggesting a link to fungal virulence^[55]^. EVs isolated from *F. graminearum* also have cytotoxic effects when infiltrated into barley leaves, but it is unknown if protein effectors, RNAs or metabolites contribute to this phenomenon^[24]^.

## RNA CONTENT

Much of the excitement surrounding EVs is related to their ability to shuttle RNA molecules between cells. Multiple studies have reported the presence of both coding and non-coding RNAs associated with mammalian EVs^[91]^. The majority of EV RNAs are small (< 200 nt), including microRNAs (miRNAs), structural RNAs and fragments of ribosomal RNAs (rRNAs), transfer RNAs (tRNAs), messenger RNAs (mRNAs) and long non-coding RNAs (lncRNAs)^[92]^. While much attention has been paid to the presence of miRNAs inside mammalian EVs as disease biomarkers and potential therapeutic molecules, miRNAs are often underrepresented in EVs compared to other species of non-coding RNAs^[91,92]^ and are enriched at higher levels in non-vesicular, extracellular particles, such as the recently defined exomeres and supermeres^[93,94]^. Other larger species of RNAs have also been detected in EV samples, including full-length mRNAs, lncRNAs and circular RNAs (circRNAs)^[95]^.

Fungal EVs are also associated with a rich assortment of RNAs. While some of these RNAs are long, including full-length mRNAs and lnRNAs, the majority are small (< 250 nt) and non-coding, including microRNA-like sequences, small RNAs (sRNAs), small nucleolar RNAs (snoRNAs), small nuclear RNAs (snRNAs), tRNAs and fragmented rRNAs^[96]^. The presence of so many non-coding RNAs (ncRNAs) suggests that fungal EVs may regulate intercellular protein synthesis and could be utilized by pathogenic fungi to interfere with host cell processes^[96]^.

Some of the most abundant species of small RNAs in fungal EVs are milRNAs^[96]^. Similar to miRNAs in plants and animals, milRNAs are typically generated from the cleavage of a precursor stem-loop RNA by Dicer-like (DCL) proteins and loaded onto an Argonaute (AGO) protein to negatively regulate the expression of mRNAs^[97]^. While miRNAs and milRNAs are similar in their biogenesis, there are at least four distinct pathways for the formation of fungal milRNAs compared to the single, canonical miRNA pathway found in plants or animals^[97]^. Additionally, milRNA precursors can be transcribed by RNA Polymerase III instead of by RNA Polymerase II, as in plants and animals^[98]^.

Small RNAs such as milRNAs are thought to promote the virulence of fungal phytopathogens. milRNAs have been predicted in multiple fungal phytopathogens, including *Sclerotinia sclerotiorum, Fusarium oxysporum, Puccinia striiformis* f. sp. *tritici, Z. tritici* and *Verticillium dahliae*^[99-104]^. These milRNAs can regulate virulence-related genes within the fungus. For example, in *V. dahliae*, the milRNA *Vd*MILR1 downregulates the essential fungal virulence factor *Vd*Hy1 through histone methylation^[102]^. Other milRNAs are predicted to enhance host susceptibility through trans-kingdom gene silencing. For example, in *Fusarium oxysporum* f. sp.* lycopersici* (Fol), *Fol*-milR is exported into tomato cells, where it enhances susceptibility to the fungus by downregulating the expression of the tomato CBL-interacting protein kinase *Sly*FRG4^[104]^. Similarly, in *Puccinia striiformis* f. sp. tritici, *Pst*-milR1 regulates the expression of predicted wheat pathogenesis-related 2 (PR2) protein and positively contributes to the virulence of the fungus^[101]^.

Only one study so far has examined the RNA contents of EVs from a phytopathogenic fungus [Table 1]. Kwon *et al*. isolated EV RNA from the biotrophic maize pathogen *U. maydis*. The study generated poly-A enriched RNA libraries from both mock- and RNase-treated EVs to rule out the presence of extra-vesicular RNAs^[57]^. The majority of EV RNAs were < 200 nt in length and likely comprised tRNAs and fragmented mRNAs and rRNAs. Due to the way in which the libraries were generated, the study did not comment on the presence of small ncRNAs, but it was able to identify several EV-enriched, full-length coding sequences. These transcripts appear to be selectively packaged into EVs and were enriched for biological gene ontology (GO) terms related to metabolic processes, proteaosomal degradation, vesicle-mediated transport, cytokinesis and iron uptake^[57]^. The study also identified transcripts for nine *bona fide* effectors as well as 161 transcripts encoding mainly metabolic enzymes that are enriched in EVs and highly expressed during the early stages of infection^[57]^. The presence of these transcripts led the authors to suggest that fungal EVs might function to reprogram plant host metabolism^[57]^.

While Kwon *et al*. remains the only study to analyze EV RNA from an axenically grown culture, a recent pre-print study by Kusch *et al*. attempted to isolate fungal RNAs from *Blumeria hordei*-infected barley plants^[57,105]^. The study isolated crude EV pellets from the apoplastic wash of infected and non-infected plants. EV pellets from infected plants were enriched for fungal milRNAs that were predicted to target barley genes more often than the endogenous fungal genes^[105]^. Predicted targets of the milRNAs were mainly protein kinases, which suggests that fungal EVs may contain milRNAs that have a role in disrupting plant immune signaling^[105]^.

Both studies suggest that coding and non-coding RNAs are packaged into the EVs of phytopathogenic fungi. Kwon *et al*. treated vesicles with RNase prior to RNA isolation and showed that significant degradation of the RNA occurred only in the presence of both RNase and a detergent to disrupt vesicles^[57]^. This suggests that the mRNAs detected are present in the EV lumen. However, extracellular RNAs can also be associated with protein complexes and lipoproteins, which would be unaffected by the presence of RNase. The study compared RNA libraries from mock- and RNase-treated EVs but did not include RNA from detergent- and RNase-treated EVs. It is therefore difficult to rule out the presence of non-vesicular protein-RNA complexes. Kusch *et al*. only analyzed crude vesicle pellets without any kind of RNase treatment. So, it is difficult to determine which detected RNAs are packaged in the EV lumen and which may be non-vesicular RNAs merely associated with the EV pellet^[105]^.

## QUESTIONS FOR THE FUTURE

The study of EVs in fungal phytopathogens is a nascent field and thus fraught with unanswered questions. Much of the work so far has focused on developing methods to isolate EVs and characterize EV content. Undoubtedly, these methods will continue to be refined. Standardization of procedures and, if possible, growth conditions for species of fungi will improve comparisons among studies. 

There is a growing appreciation of the heterogeneity of fungal EVs. Evidence from *A. infectoria* suggests there are two separate populations of differently sized EVs^[60]^, while EVs from *C. higginsianum* and *F. graminearum *are separated into two distinct populations on the basis of buoyant density^[24,59]^. These separate populations suggest there may be multiple distinct pathways for EV biogenesis, but this has not yet been shown. Most studies analyze the EV pellet *in toto*, meaning current proteomes and RNA libraries reflect a mixture of different vesicle populations and co-pelleting protein complexes. Greater care should be taken to purify vesicles using methods such as SEC and iodixanol density-gradients and to verify the packaging of proteins and RNAs inside EVs; this means treating EVs with protease or protease followed by RNase to confirm EV protein or RNA cargo, respectively.

Fungal EVs have been almost exclusively isolated from cultures grown in liquid or on solid medium. Apart from the pre-print Kusch *et al*., no other study has attempted to isolate fungal EVs from an infected organism^[105]^. Because growth conditions affect the contents and composition of EVs, it is probable that EVs from fungal phytopathogens infecting plants may contain different cargo compared to those isolated from axenically grown fungi^[62,64]^. Such cargo may better reflect the virulence-promoting activities of fungal EVs and could lead to the discovery of new effectors or toxic metabolites. However, detecting EV protein or RNA signals in a sample mainly containing plant materials will prove challenging and may require the development of immunoprecipitation protocols to separate fungal EVs. Additionally, infection structures produced by an invading fungal phytopathogen (e.g., haustoria) are usually encased in callose and separated from the rest of the plant apoplast by a collar around the haustoria neck^[106]^. Such collars would limit the diffusion of EVs from the fungal extra-haustorial space into the bulk plant apoplast, potentially stymying attempts to isolate fungal EVs from plant apoplastic fluids^[22]^. Until better methods are developed, researchers will have to rely on growth media that mimic the environment of the plant and stimulate fungal virulence. In this manner, it may be possible to examine fungal EVs that are more similar to those produced during infection.

Another set of long-standing questions involves the production and secretion of fungal EVs. A few studies have successfully identified genetic components that contribute to EV formation and secretion in yeasts. Mutations in the *SEC6* gene of *C. neoformans*, which encodes a subunit of the exocyst complex, compromises EV secretion^[107]^. In *S. cerevisiae*, mutations in several different genes encoding different regulators of endomembrane trafficking have been shown to affect EV size and composition. These include *SEC4*, which encodes a vesicle associated RabGTPase, Golgi Reasssembly Stacking Protein (GRASP; a regulator of unconventional secretion), and several members of the ESCRT complex^[86,108]^. Lipid transporters also affect EV sizes in *Cryptococcus neoformans* and *C. gattii*^[109,110]^. *C higginsianum, Fov, F. graminearum U. maydis *and *Z. tritici* are all genetically tractable organisms, so it is feasible that orthologous candidates of EV biogenesis and secretion in yeasts could be evaluated in fungal phytopathogens^[111-115]^.

When and where EVs are secreted from fungal phytopathogens are also important questions to be answered. Unlike yeasts, filamentous fungal plant pathogens develop different cell types throughout an infection, including germ tubes, surface hyphae, appressoria, penetration pegs and haustoria/infection hyphae. EVs could be secreted from any of these cell types. EVs can be secreted from vegetative hyphae, so it is possible that infection hyphae within plant tissues also secrete EVs. The question then is “which regions of the hyphae secrete vesicles?” Hyphal tips are sites of active exocytosis, but secretory events can also occur in regions distal to the tip^[116]^. Based on the localization of fluorescent EV markers in *C. higginsianum*, EV secretion may occur at the tips of infection hyphae, but this has yet to be verified^[59]^.

Last but not least, functionality remains an elusive question in most EV studies. Researchers can predict the roles and activities of EVs based on their cargo, but experimentally verifying a function is another matter. While fungal phytopathogen EVs putatively promote virulence, only two studies have demonstrated a direct cytotoxic effect of fungal EVs in leaves^[24,52]^. It will be interesting to see if EVs from other fungal phytopathogens can also induce plant cell death or produce other predicted effects, such as altering host cell metabolism or suppressing defense responses.

## CONCLUSIONS

Phytopathogenic fungi pose a severe threat both ecologically and to global food security. Estimates suggest that fungi destroy a third of all food crops annually, leading to massive human suffering and economic loss^[26]^. Understanding how fungi infect plants is an important step toward developing better systems of disease management and engineering more robust crops. Emerging evidence suggests that EVs from fungal plant pathogens are important mediators of virulence, capable of transporting protein effectors, toxic secondary metabolites and RNA^[52,55,57,59,60,62]^. While research into fungal phytopathogen EVs is progressing quickly, there is still much work to be done. So far, significant efforts have been made to characterize EVs secreted by multiple pathogens. Standardized methods for fungal growth and EV isolation, as well as greater inclusion of tests to validate EV cargo, will help clarify EV composition and lead to the development of important markers for vesicle tracking and isolation. As researchers gain a better understanding of the composition of fungal phytopathogen EVs, it will be necessary to use these data to address questions of EV biogenesis, secretion and diversity and to test predicted functions *in planta*. A thorough understanding of the biogenesis and function of EVs in phytopathogenic fungi may lead to the development of new fungicides or crop-based technologies to block the production of fungal EVs or impair their functions in hosts.

## DECLARATIONS

### Copyright

© The Author(s) 2023.
